# Compositional distinction of gut microbiota between Han Chinese and Tibetan populations with liver cirrhosis

**DOI:** 10.7717/peerj.12142

**Published:** 2021-09-15

**Authors:** Hui Huan, Tao Ren, Li Xu, Hong Hu, Chao Liu

**Affiliations:** Hospital of Chengdu Office of People’s Government of Tibetan Autonomous Region, Chengdu, China

**Keywords:** Tibetan population, Liver cirrhosis, Gut microbiota, 16S rRNA gene sequencing

## Abstract

**Background:**

Liver cirrhosis (LC) is caused by numerous chronic liver diseases and its complications are associated with qualitative and quantitative alterations of the gut microbiota. Previous studies have revealed the characteristics of gut microbiota in Han Chinese patients with LC and different compositions of gut microbiota were reported between the Tibetan and Han Chinese populations. This study was designed to evaluate the unique features of the gut microbiota of Tibetans and compare the differences of gut microbiota between Tibetan and Han Chinese patients with LC.

**Methods:**

Thirty-six patients with liver cirrhosis and nineteen healthy volunteers, from both Tibetan and Han Chinese populations, were enrolled and fecal samples were collected for 16S rRNA gene sequencing analyses.

**Results:**

Significant differences were found in the gut microbiota of healthy volunteers and between Tibetan and Han Chinese patients with LC. In the Han Chinese patients with cirrhosis (HLC) group the relative abundances of the phylum *Bacteroidetes* was significantly reduced (*P* < 0.001), whereas in the Tibetan patients with cirrhosis (TLC) group *Firmicutes* and *Actinobacteria* were highly enriched (*P* = 0.01 and 0.03, respectively). At the genus level, the relative abundances of *Anaerostipes* (*P* < 0.001), *Bifidobacterium* (*P* = 0.03), and *Blautia* (*P* = 0.004) were prevalent, while *Alloprevotella*, *Dorea*, *Prevotella_2*, *Prevotella_7* and *Prevotella_9* were decreased in the TLC group compared to the HLC group (*P* < 0.01).

**Conclusion:**

Our findings showed how the intestinal bacterial community shifted in Tibetan patients with cirrhosis.

## Introduction

Liver cirrhosis, characterized by a disruption of hepatic architecture and portal hypertension, is the pathologic end stage of a wide range of acute or chronic liver injuries, including hepatitis virus infection, alcohol abuse and obesity (Qin et al., 2014; Sanduzzi et al., 2017). Currently, 4% to 10% of the population worldwide suffers from liver cirrhosis (Łapiński & Łapińska, 2019). Patients with decompensated liver cirrhosis often have a poor prognosis and eventually require liver transplants (Qin et al., 2014). Chung et al. (2018)’s study found that the relative mortality of liver cirrhosis was higher than that of five major cancers (lung, colorectal, stomach, liver, and breast cancers). Although some research has been done on the pathogenesis of liver cirrhosis (Albrich & Hefel, 2014), more work is needed to gain a better understanding of this disease.

There is a strong relationship between liver cirrhosis and gut microbiota because of the gut-liver axis, which links the liver and gut through the hepatic portal (Acharya & Bajaj, 2017; Bolger, Lohse & Usadel, 2014; Valerio et al., 2014). The coevolution of gut microbiota and human hosts has led to physiological homeostasis (Hooks & O’Malley, 2017). In a healthy human body, the intestinal barrier and liver detoxification function prevents the translocation of the gut microbiota that help to maintain the homeostasis of the intestinal microbiota constitution. However, in liver cirrhosis patients, impaired liver function results in the decrease of pancreato-biliary secretions, the diminution of intestine peristalsis, and the increase of intestinal permeability. All this further influences the normal structure of gut microbiota, or even leads to enteric dysbacteriosis and bacterial translocation (Hiroshi, 2015; Zeuzem, 2000). The alterations of intestinal microbiota have been reported to promote the progression of liver cirrhosis and the development of serious complications, such as hepatic encephalopathy (HE), variceal bleeding, spontaneous bacterial peritonitis (SBP), ascites, and other manifestations of volume overload (Acharya & Bajaj, 2017). Therefore, gut microbiota has been taken into account as a therapeutic target of liver cirrhosis; probiotics, prebiotics, and antibiotics have all been used for the clinical treatment and amelioration of this disease (Li et al., 2015; Qamar, 2015; Woodhouse et al., 2018).

Previous studies have verified distinct microbial community shifts in liver cirrhosis patients in comparison with healthy controls (Qin et al., 2014; Yanfei et al., 2011). However, there are many factors that can influence the composition of gut microbiota. A number of researchers have suggested that ethnic origin contributes to the shaping of the human gut microbial community (Ishikawa et al., 2013; Lai-Yu et al., 2014; Li et al., 2016). Native Tibetans that live on the Qinghai-Tibet plateau, characterized by high radiation, low oxygen levels, and reduced air pressure (Leónvelarde et al., 2005), have adapted to this extreme environment, by developing special dietary habits, lifestyles, and genetic predispositions (Dan et al., 2013). The Tibetans’ diet, which consists principally of beef, Yak butter, and mutton with few vegetables and fruits, could be a major influence on their gut microbiome (Liu, 2012). Therefore, it remains to be seen whether there is cross-applicability of treatments from HLC patients to the Tibetan population.

In this study, we aimed to reveal the characteristics of the intestinal microbiota constitution in the Tibetan population with liver cirrhosis using 16S rRNA gene sequencing and to understand the similarities and differences of microbial communities between Han Chinese and Tibetan patients with liver cirrhosis. This work is intended to expand the knowledge of the gut microbiota in the Tibetan population with liver cirrhosis and provide a more comprehensive understanding of the role of gut microbiota in liver cirrhosis.

## Materials & Methods

### Ethics statement

All individuals were recruited from the Department of Digestive Medicine, Hospital of Chengdu Office of People’s Government of the Tibetan Autonomous Region. The study was approved by the Medical Ethics Committee of the Hospital of Chengdu Office of People’s Government of Tibetan Autonomous Region in Chengdu, China (approval number: (2017) study No. 10). Written informed consent was obtained from each subject in this study. Inclusion criteria: patients with an identified risk factor for cirrhosis; portal hypertension and liver dysfunction; evidence of LC on abdominal CT or color doppler ultrasound imaging (Acharya & Bajaj, 2017; Acharya & Bajaj, 2019).

### Human subjects

A total of 55 individuals were divided into four groups: Tibetan healthy control (THC) group (*n* = 10), Han Chinese healthy control (HHC) group (*n* = 9), Tibetan patients with LC group (*n* = 20), and Han Chinese patients with LC group (*n* = 16).

### Fecal sample collection and genomic DNA extraction

Fecal samples of all the subjects in this study were collected and immediately frozen at −80 °C for further analyses. Total genomic DNA was extracted from the frozen fecal samples using the DNeasy PowerSoil Kit (Tiangen Biotechnology Company, Beijing, China) following the manufacturer’s instructions. Quality and quantity of DNA were verified with the NanoDrop spectrophotometer and agarose gel. Extracted DNA was diluted to a concentration of one ng/μl and stored at −20 °C until further processing.

### 16S rRNA gene analysis of fecal samples

The 16S rRNA gene analysis of the fecal samples was performed as previously described (Qiu et al., 2019). Briefly, the diluted DNA obtained above was used as a template for polymerase chain reaction (PCR) amplification of the bacterial 16S rRNA gene with V3-V4 region-specific primers: 343F (5′-TACGGRAGGCAGCAG-3′) and 798R (5′-AGGGTATCTAATCCT-3′). An Illumina sequencing adaptor was added to the primers and the reverse primer contained a sample barcode. The amplicon quality was visualized using gel electrophoresis before the PCR products were purified. Next, concentrations were adjusted for sequencing on an Illumina MiSeq PE300 system (OE Biotech Co., Ltd, Shanghai, China).

### Data analyses

Bioinformatics analyses were performed as previously described (Derakhshani et al., 2016). Raw sequencing data was generated in the FASTQ format. Paired-end reads were preprocessed using Trimmomatic (v0.35) (Bolger, Lohse & Usadel, 2014) to detect and cut off ambiguous bases Low quality sequences were cut off using the sliding window trimming approach and the paired-end reads were assembled using FLASH (v1.2.11) (Reyon et al., 2012). Then using QIIME (v1.8.0) (Caporaso et al., 2010) and UCHIME (v4.2) (Edgar et al., 2011), the sequences were denoised as follows: reads with ambiguous sequences, homologous sequences, or below 200bp were abandoned and reads with 75% of bases above Q20 were retained. All sequences with mismatches or ambiguous calls in the overlapping region were discarded. Clean reads were subjected to primer sequences removal and clustering to generate operational taxonomic units (OTUs) using VSEARCH (v2.4.2) (Rognes et al., 2016) software with a 97% similarity cutoff (Edgar, 2013). All representative reads were annotated and blasted against the SILVA database (v123) (or Greengenes) (16srDNA) using the RDP classifier (v2.2) (confidence threshold was 70%) (Wang et al., 2007). The phylotypes were computed as percent proportions based on the total number of sequences in each sample. Alpha- and beta-diversity indexes were calculated using QIIME as previously described (Bindels et al., 2016). To visualize α diversity, we employed QIIME software to calculate α estimators for each sample including the Shannon index, Chao1 index, Simpson’s Diversity Index, and Observed Species. In addition, β diversity between the communities was assessed by weighted UniFrac for Principal Coordinate Analysis (PCoA).

### Statistical analysis

Statistical analyses were performed using SPSS software version 20.0 (SPSS Inc., Armonk, NY, USA) and GraphPad Prism (V.6.0). Principal coordinate analysis (PCoA) was applied on the resulting distance matrices to generate plots using the default settings of the PRIMER-6 software (Derakhshani et al., 2016). Comparison of OTUs and taxonomy abundances was calculated using the Kruskall–Wallis test or Mann Whitney analysis. Following statistical analyses with multiple comparisons, *P* values were corrected using the Benjamini–Hochberg method to control the false discovery rate (FDR) (Freedberg et al., 2015). The resultant *P-*values were FDR corrected with a significance threshold of 5%. Furthermore, the linear discriminant analysis (LDA) and the linear discriminant analysis effect size (LEfSe) measurements were used to find unique bacterial taxa among different groups (Segata et al., 2011) (LDA > 2, FDR-*p* < 0.05) and *P*-values < 0.05 were considered statistically significant.

## Results

### Community richness and biodiversity

**Figure 1:**
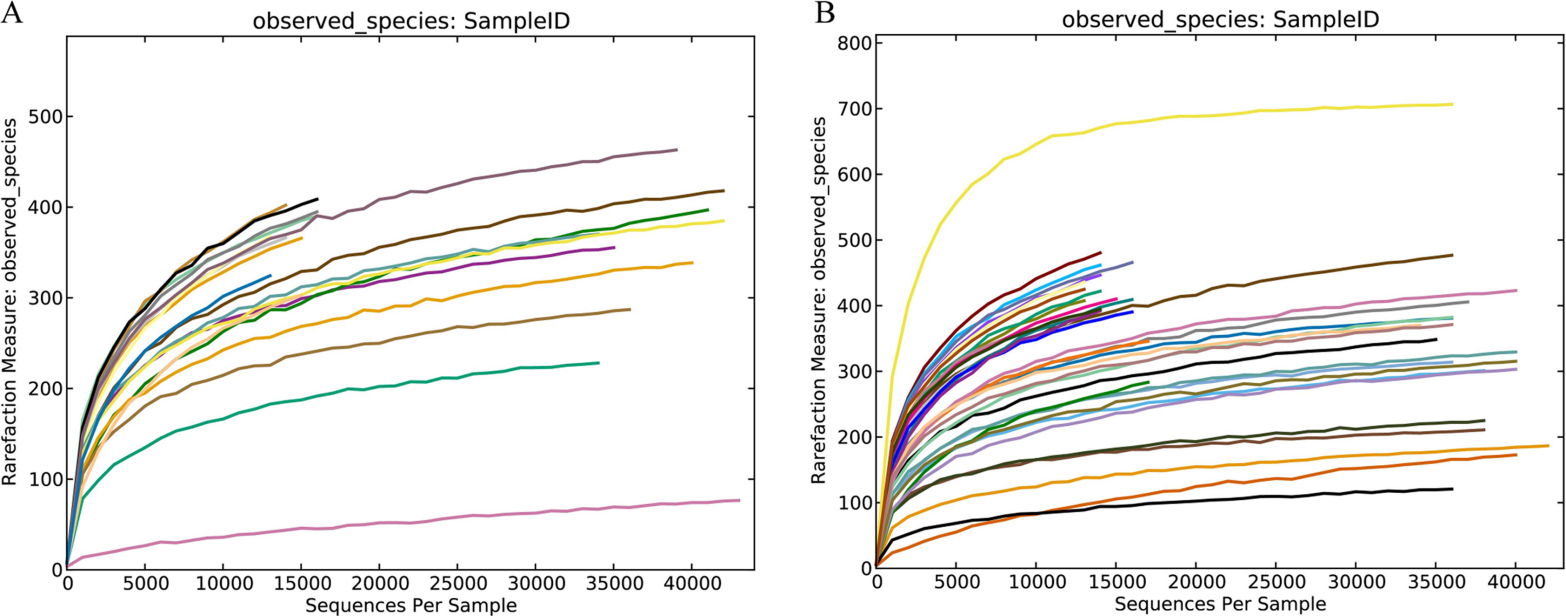
Community richness and biodiversity of healthy subjects and patients with liver cirrhosis. Rarefaction curves of the healthy volunteers (A) and patients with liver cirrhosis (B).

In this study, the related characteristics of patients and healthy controls (Table 1) indicated that there was no difference in age, sex, and BMI (body mass index). A total of 55 fecal samples were collected from the four groups and underwent PCR amplification of the V3-V4 hypervariable regions of the bacterial 16S rRNA gene. Raw data for the 16S rRNA gene has been deposited in the NCBI SRA database with the accession number PRJNA603295. After filtration by QIIME quality filters an average of 50,770, 19,914, 48,338 and 19,963 clean tags were obtained from the THC, HHC, TLC and HLC groups, respectively and among these clean tags, an average of 39,053 (76.92%), 15,143 (76.04%), 36,872 (76.28%) and 15,006 (75.17%) tags were valid in their respective groups (Table S1). The lengths of all the valid tags were 428 ~ 431 bp (Table S1). All valid tags were clustered to operational taxonomic units (OTUs) using VSEARCH software at a 97% similarity threshold and a total of 1,579 unique OTUs were generated. Rarefaction curves of the healthy and liver cirrhosis humans displaying the richness and evenness of the gut microbiota in the samples can be found in Fig. 1. As the sequences increased, the amounts of Observed Species gradually stabilized. Additionally, the Good’s coverage indexes were over 99% in all samples (Table S2). These results collectively indicated that sequencing depth in our study was sufficient, and the sequencing results were reasonable and reliable enough for further analyses.

**Table 1:** Patient and healthy control characteristics.

Characteristics	Ages, years*	Sex, male/female	Body mass index*
THC (*n* = 10)	48.7 ± 10.01	6/4	23.21 ± 0.87
HHC (*n* = 9)	48.33 ± 11.07	6/3	23.94 ± 1.75
TLC (*n* = 20)	51.2 ± 11.18	16/4	22.95 ± 2.12
HLC (*n* = 16)	49.18 ± 10.91	13/3	22.81 ± 2.51

**Note:**

* Data are expressed as the mean ± SD.

### Compositional distinction of gut microbiota of all the groups between Han Chinese and Tibetan populations

**Figure 2:**
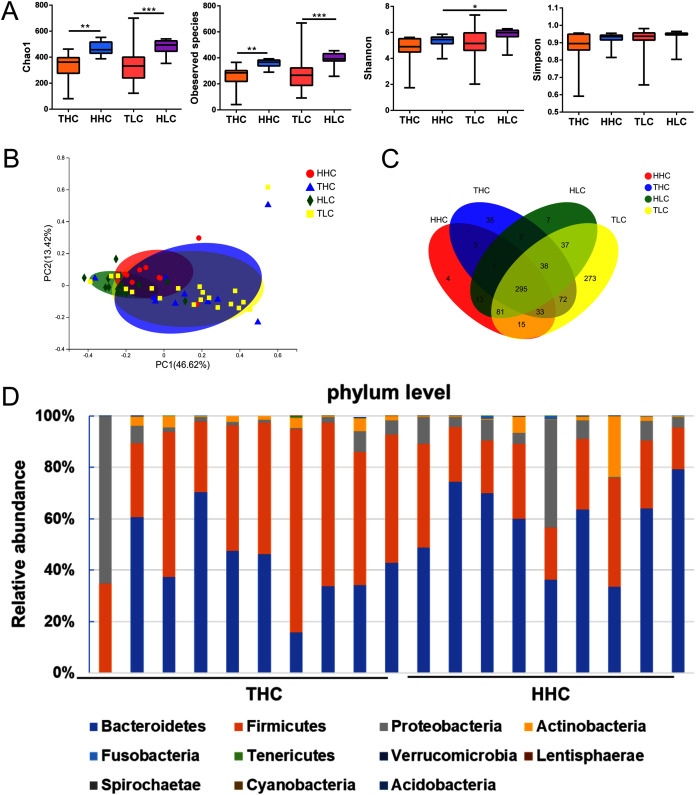
The α diversity and β diversity of fecal microbiota of four groups. (A) αdiversity (ANOVA analysis); (B) principal coordinate analysis plot of unweighted UniFrac distances (Adnonis analysis); (C) the Venn diagram of four groups; (D) composition and the dominant taxa at the phylum level between HHC and THC group. THC, healthy Tibetans group; HHC, healthy Han volunteers group; TLC, Tibetans with liver cirrhosis; HLC, the Chinese Han with liver cirrhosis. *P < 0.05, **P < 0.01, ***P < 0.001.

After obtaining valid tags, the α diversity and β diversity metrics showed differences among the four groups. In general, the richness of α diversity, as determined by the Chao1 index or Observed Species, was significantly higher in Han Chinese populations compared to Tibetan populations (*P* < 0.01), while no significance was found in either the Shannon index or Simpson’s Diversity Index representing diversity and community evenness (*P* > 0.05). In the HHC group, the Chao1 index and Observed Species were higher compared to the THC group (*P* = 0.0015 and *P* = 0.0019 respectively); this trend was similar in patients with LC. In the HLC group, the Chao1 index or Observed Species were significantly higher than that of the TLC group (*P* = 0.0006 and *P* = 0.0001 respectively). As for β diversity analysis, in weighted UniFrac PCoA the first principal coordinate (PC1) occupied 41.92% of inter sample variance and revealed that inter-individual variation is greater than intra-individual variation, indicating a clear distinction between the gut microbiota of the THC and HHC groups (*P* = 0.011) (Fig. 2B). These findings suggested that ethnic factors have an important influence on intestinal flora.

The V3–V4 sequences from the THC and the HHC groups were predominately assigned to eleven phyla: *Bacteroidetes*, *Firmicutes*, *Proteobacteria*, *Actinobacteria*, *Fusobacteria*, *Tenericutes*, *Verrucomicrobia*, *Lentisphaerae*, *Spirochaetae*, *Cyanobacteria* and *Acidobacteria*; the relative abundance bar plot of the gut microbiota community at the phylum level was displayed in Fig. 2D. The results showed that the dominant phyla were *Firmicutes* and *Bacteroidetes* in both the THC and the HHC groups. Collectively, these results suggested that there was an ethnicity-based diversity of gut microbiota between Tibetan and Han Chinese populations as previously reported (Lai-Yu et al., 2014).

**Figure 3:**
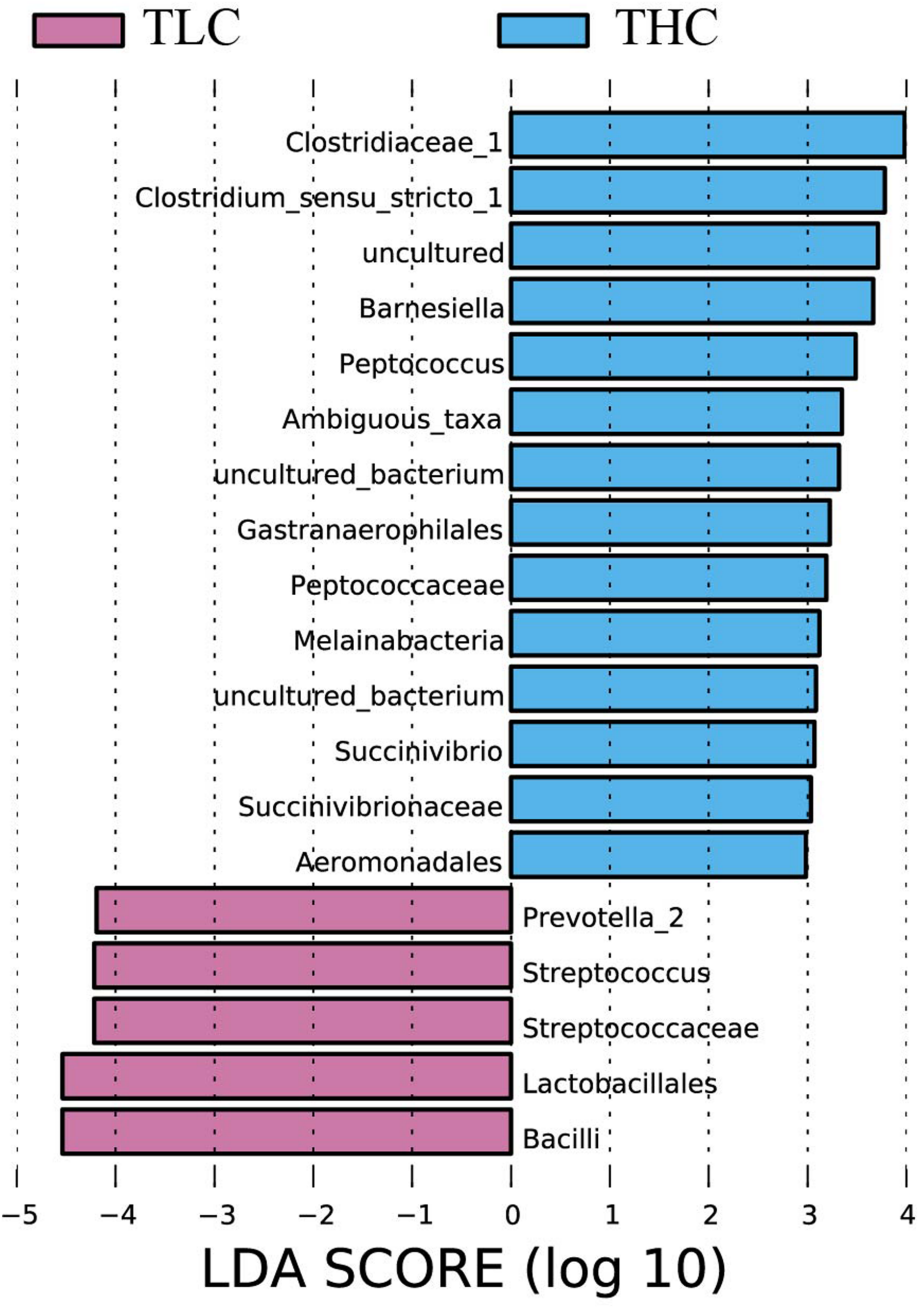
The linear discriminant analysis effect size analysis of fecal microbiota between Tibetan healthy population and Tibetan patients with liver cirrhosis.

### Identification of gut microbiota significantly varied in Tibetan populations with liver cirrhosis patients

LEfSe analysis of taxon abundance was conducted to detect the differences in fecal bacteria between Tibetan patients with LC and Han Chinese patients with LC. Thus, the microbial signature in both two groups was identified. The relative abundances of *Clostridiaceae_1*, *Clostridium_sensu_stricto_1*, *Barnesiella*, *Peptococcus*, *Ambiguous_taxa*, *Gastranaerophilales*, *Peptococcaceae*, *Melainabacteria*, *Succinivibrio*, *Succinivibrionaceae*, and *Aeromonadales* as well as some uncultured bacterium were more prevalent in Tibetan healthy populations than t in Tibetan patients with liver cirrhosis (Fig. 3). However, more *Prevotella_2*, *Streptococcus*, *Streptococcaceae*, *Lactobacillales*, and *Bacilli* were found in Tibetan patients with liver cirrhosis (Fig. 3). Among these bacteria, *Streptococcaceae* has been reported to be prevalent in patients with cirrhosis (Yanfei et al., 2011). In addition, the LEfSe analysis between cirrhosis patients from both ethnic groups is in Fig. S1.

### Comparison of gut microbiota between Han Chinese and Tibetan populations with liver cirrhosis

**Figure 4:**
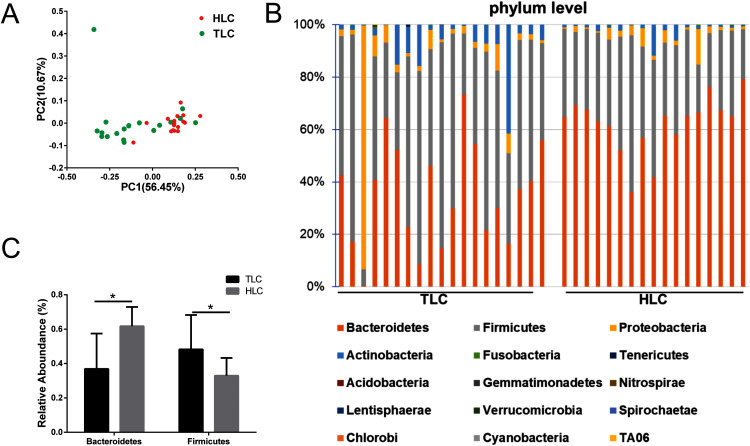
The differences of fecal microbiota between TLC and HLC groups at the phylum level. (A) Principal coordinate analysis plot of unweighted UniFrac distances. (B) Composition and relative abundance communities (top 15) at the phylum level. (C) The relative abundances of *Bacteroidetes, Firmicutes* between TLC and HLC groups. Kruskal–Wallis analysis, *P < 0.05.

We investigated the microbial communities of Han Chinese and Tibetan patients with liver cirrhosis to explore whether different microbial composition exists between these two groups. The information of all the diversity indexes for Tibetan and Han Chinese patients with liver cirrhosis is in Table S2. As a result, the PCoA plots showed a clear separation between the HLC and TLC groups on the graph (Fig. 4A). The percentages of variation explained by PC1 and PC2 were 56.45% and 10.67%. Two distinct areas from samples of two groups on the graph indicated a significant difference between the gut bacterial compositions. All taxonomic assignments and their abundances are in Table S3.

Then, we analyzed gut microbiota composition at the phylum level in both groups and the relative abundance chart is in Fig. 4B. The top 15 most abundant bacteria in phylum level were *Bacteroidetes*, *Firmicutes*, *Proteobacteria*, *Actinobacteria*, *Fusobacteria*, *Tenericutes*, *Acidobacteria*, *Gemmatimonadetes*, *Nitrospirae*, *Lentisphaerae*, *Verrucomicrobia*, *Spirochaetae*, *Chlorobi*, *Cyanobacteria* and *TA06*, seven of which were in accordance with previous studies (Yanfei et al., 2011). Additionally, the relative abundance of *Bacteroidetes* and *Firmicutes* at the phylum level were significantly different between the TLC and HLC groups respectively (*P* < 0.001 and *P* = 0.008, Fig. 4C).

**Figure 5:**
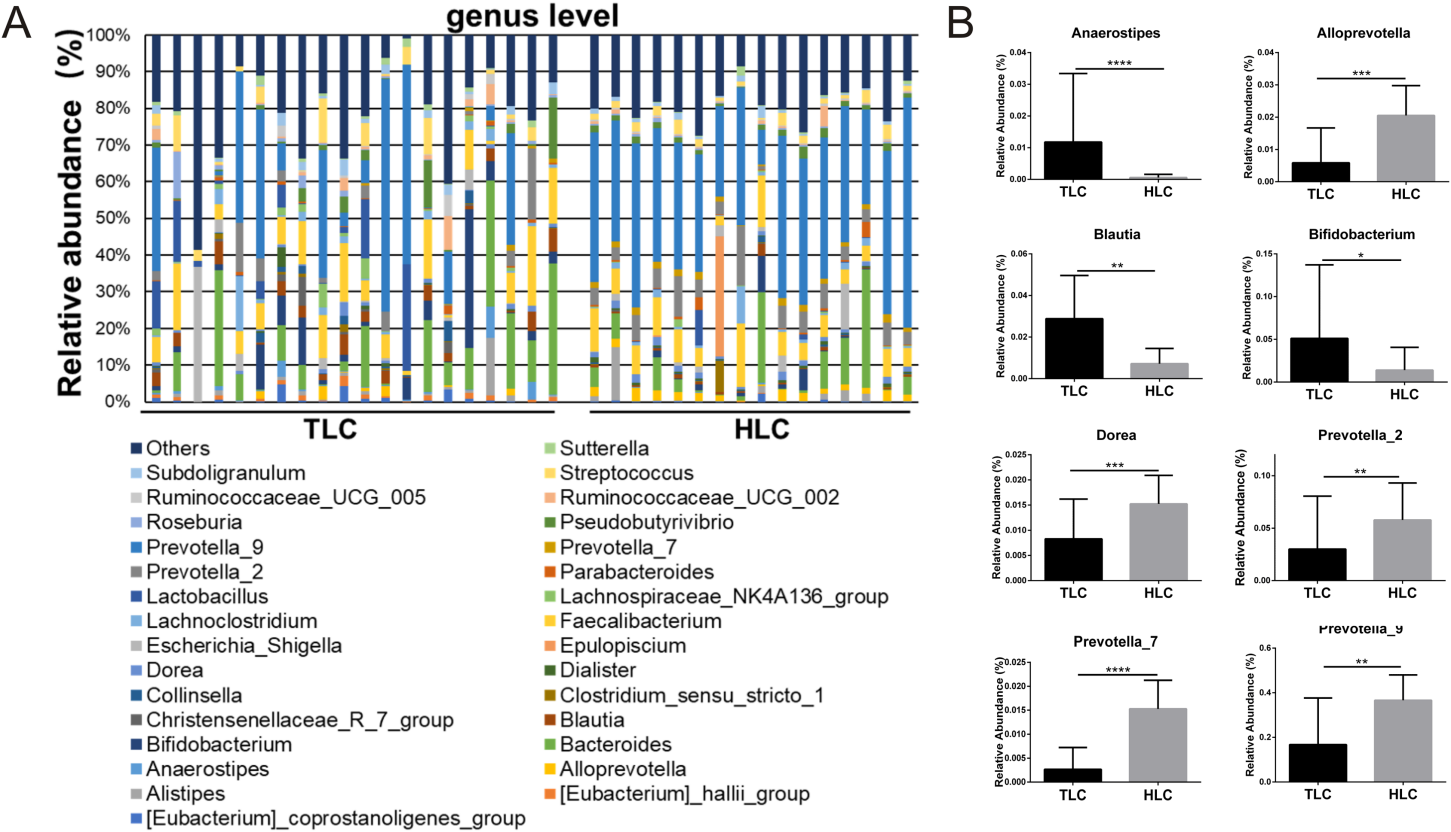
The differences of fecal microbiota between TLC and HLC groups at the genus level. (A) Composition and relative abundance communities of top 30 taxa at the genus level. (B) The relative abundance of representative fecal bacterial between TLC and HLC groups at the genus level. Kruskal–Wallis analysis, *P < 0.05, **P < 0.01, ***P < 0.001, ****P < 0.0001.

Then, a percentage stacked histogram was used to display the relative abundance of gut microbiota at the genus level; the top 30 most abundant bacteria at the genus level are shown in Fig. 5A. In both the TLC and HLC groups, *Prevotella_9* was the predominant genus. In total, eight significantly different pairs were found in *Alloprevotella* (*P* < 0.001), *Anaerostipes* (*P* < 0.001), *Bifidobacterium* (*P* = 0.03), *Blautia* (*P* = 0.0035), *Dorea* (*P* =<0.001), *Prevotella_2* (*P* = 0.0015), *Prevotella_7* (*P* < 0.001) and *Prevotella_9* (*P* = 0.0028). In the TLC group, the relative abundance of *Anaerostipes*, *Bifidobacterium*, and *Blautia* was higher than in the HLC group (Fig. 5B). However, the relative abundance of *Alloprevotella, Dorea*, *Prevotella_2*, *Prevotella_7*, and *Prevotella_9* were decreased in the TLC group. Taken together, these results indicated a remarkable difference between Tibetan and the Han Chinese patients with LC in terms of constituent gut microbiota, suggesting the potential influence of ethnic and environmental factors in the development of LC.

## Discussion

The relationship between the gut microbiota and the liver is becoming increasingly important to the scientific community. The gut-liver axis is relevant to the development of liver cirrhosis and its complications and many studies have indicated that gut microbiota is a potential target for the treatment and modulation of liver diseases (Acharya & Bajaj, 2019; Milosevic et al., 2019). Gut microbiota is a diverse microbial ecosystem and can be affected by various factors, such as age, diet, medications, environment, and ethnics (Brooks et al., 2018; Deschasaux et al., 2018; Milosevic et al., 2019; Wu et al., 2011). Some researchers have reported that there are discriminating microbiota compositions between the Tibetans that live on the Qinghai-Tibet Plateau for generations and the Han Chinese populations (Li et al., 2016; Li & Zhao, 2015). Thus, even though some researchers have reported on the gut microbiome of the Han Chinese patients with LC, it is still necessary to study the composition of the gut microbiota in Tibetan populations with LC. In this study, 16S rRNA gene sequencing technology was used to characterize the fecal microbiota of Tibetans with LC and compared it to both healthy individuals and the Han Chinese patients with LC.

In our study, we first compared the α and β diversity among the four groups and observed distinctions that were in accordance with previously reported results (Li et al., 2016; Li & Zhao, 2015); this verified that ethnic factors are indeed involved in shaping the gut microbiota of human beings. The Chao1 index significantly declined in the THC group, which indicated that there was less gut microbiota in the THC group. Consistently, Observed Species (one of the α-diversity indexes) was significantly lower in the THC group than in the HHC group.

Alterations of gut microbiota in the Tibetan liver cirrhosis patients were investigated in comparison with healthy Tibetans. As reported here, the following bacteria, found using LEfSe analysis, were prevalent in the THC group: *Clostridiaceae_1*, *Clostridium_sensu_stricto_1*, *Barnesiella*, *Peptococcus*, *Ambiguous_taxa*, *Gastranaerophilales*, *Peptococcaceae*, *Melainabacteria*, *Succinivibrio*, *Succinivibrionaceae*, and *Aeromonadales*. In contrast, *Prevotella_2*, *Streptococcus*, *Streptococcaceae*, *Lactobacillales*. and *Bacilli* were significantly elevated in Tibetan patients with LC as previously reported (Ahluwalia et al., 2016b; Bajaj et al., 2015; Chen et al., 2016; Qin et al., 2014; Riordan & Williams, 2006; Wei et al., 2013; Yanfei et al., 2011). Elevated *Streptococcus* (class *Bacilli*) is prevalent in bacterial infections in LC patients (Qin et al., 2014; Riordan & Williams, 2006; Wei et al., 2013; Yanfei et al., 2011). The overabundance of microbes, such as *Prevotella*, may play an important role in bacteria translocation in cirrhosis by producing signaling microbial metabolites that may influence and initiate the inflammatory mediators that lead to liver inflammation and cirrhosis (Chen et al., 2011; Niederau et al., 1998). As for *Prevotellaceae*, it is regarded as a possible bacterial translocation in Tibetans with LC. In previous articles, cirrhotic patients with hepatic encephalopathy showed a higher abundance pattern of *Lactobacillaceae*, which are positively related with ammonia, model for end-stage liver disease (MELD) score, and brain MR spectroscopy (MRS) manifestations (Ahluwalia et al., 2016a). These results were consistent with previous research on Han Chinese patients with liver cirrhosis.

Subsequently, our emphasis was on exploring the differences between Han Chinese and Tibetan populations with liver cirrhosis at the phylum and genus levels. At the phylum level, the relative abundance of *Firmicutes* was significantly higher, whereas the proportion of *Bacteroidetes* was lower in Tibetan patients with cirrhosis. The Firmicutes/Bacteroidetes (F/B) ratio is widely accepted to have an important influence in maintaining normal intestinal homeostasis. Increased or decreased F/B ratio is regarded as dysbiosis (Stojanov, Berlec & Štrukelj, 2020). This might indicate that Tibetans with cirrhosis have a higher risk to develop different complications. The analysis at the genus levels revealed the prevalence of various bacteria in the TLC group, including *Anaerostipes*, *Bifidobacterium*, and *Blautia*, whereas the relative abundance of *Alloprevotella, Dorea*, *Prevotella_2*, *Prevotella_7* and *Prevotella_9* decreased in Tibetan cirrhosis patients. Among these genera, *Dorea* has been suggested to be potentially beneficial and associated with the reduction of inflammation in liver cirrhosis patients (Wu et al., 2011). The decrease of *Dorea* in the TLC group showed a potentially higher risk to develop complications such as HE, which was consistent with the results mentioned above. *Blautia*, as a 7α-dehydroxylating bacteria, belongs to the potentially pathogenic family *Enterobacteriaceae* (Kakiyama et al., 2013). The high level of *Prevotella* was related to the high intake of carbohydrates and simple sugars (Wu et al., 2011). Tibetan dietary habits are dominated by meat such as beef and mutton, fermented dairy products such as yogurt, and a lack of carbohydrates, vegetables, and fruits (Li et al., 2016), so it was reasonable that the abundance of *Prevotella* was lower in Tibetan liver cirrhosis patients than in Han Chinese liver cirrhosis patients. *Prevotella* is generally presented in the oral cavity and involved in the production of high levels of CH_3_SH and CH_3_SH in blood is considered an important factor in pathogenesis of HE (Bik et al., 2010; Al Mardini, Bartlett & Record, 1984; Takeshita et al., 2012). This might be relevant to the development of HE in Han Chinese patients with liver cirrhosis. Additionally, the elevation of *Bifidobacterium* appeared to be associated with the severity of liver injury (Lu et al., 2011). Thus, these results demonstrated that the mechanisms of complications’ development were potentially different between Han Chinese and Tibetan patients with liver cirrhosis, suggesting that individual-based regulation of gut microbiota in liver cirrhosis patients is required for different ethnicities. However, further studies with larger sample sizes and more subgroup analyses to exclude the effects of other factors such as dietary habits are needed to confirm these speculations.

## Conclusions

In summary, this study characterized the fecal microbial communities in Tibetan patients with liver cirrhosis using 16S rRNA gene sequencing technology and presented the comparison between Tibetan liver cirrhosis patients, healthy individuals, and Han Chinese liver cirrhosis patients. The results showed that the intestinal microbiota composition of Tibetan patients with cirrhosis was different from the controls, and this trend was also found between Tibetan and Han Chinese patients with cirrhosis. Additionally, differing abundance of gut microbiota between Tibetan and Han Chinese patients with LC were observed, providing a more comprehensive understanding of fecal microbiota in Tibetans with LC and this may provide guidance for further research on individual treatments for cirrhosis in the Tibetan populations.

## Supplemental Information

10.7717/peerj.12142/supp-1 Supplemental Information 1 Analysis of differences in the microbiota between Tibetan and Chinese Han patients with liver cirrhosis using Lefse method.

10.7717/peerj.12142/supp-2 Supplemental Information 2 Sequencing Data Summary.

10.7717/peerj.12142/supp-3 Supplemental Information 3 α-diversity of gut microbiota in faeces samples from all sample.

10.7717/peerj.12142/supp-4 Supplemental Information 4 OTU abundance of each sample.

10.7717/peerj.12142/supp-5 Supplemental Information 5 Raw data of Han healthy control group including HHC1-HHC5.

10.7717/peerj.12142/supp-6 Supplemental Information 6 Raw data of Han healthy control group including HHC6-HHC9.

10.7717/peerj.12142/supp-7 Supplemental Information 7 Raw data of Han with liver cirrhosis group including HLC1-HLC5.

10.7717/peerj.12142/supp-8 Supplemental Information 8 Raw data of Han with liver cirrhosis group including HLC6-HLC10.

10.7717/peerj.12142/supp-9 Supplemental Information 9 Raw data of Han with liver cirrhosis group including HLC11-HLC16.

10.7717/peerj.12142/supp-10 Supplemental Information 10 Raw data of Tibetan healthy control group including THC1-THC2.

10.7717/peerj.12142/supp-11 Supplemental Information 11 Raw data of Tibetan healthy control group including THC3-THC4.

10.7717/peerj.12142/supp-12 Supplemental Information 12 Raw data of Tibetan healthy control group including THC5-THC6.

10.7717/peerj.12142/supp-13 Supplemental Information 13 Raw data of Tibetan healthy control group including THC7-THC8.

10.7717/peerj.12142/supp-14 Supplemental Information 14 Raw data of Tibetan healthy control group including THC9-THC10.

10.7717/peerj.12142/supp-15 Supplemental Information 15 Raw data of Tibetan with liver cirrhosis group including TLC1-TLC3.

10.7717/peerj.12142/supp-16 Supplemental Information 16 Raw data of Tibetan with liver cirrhosis group including TLC4-TLC6.

10.7717/peerj.12142/supp-17 Supplemental Information 17 Raw data of Tibetan with liver cirrhosis group including TLC7-TLC8.

10.7717/peerj.12142/supp-18 Supplemental Information 18 Raw data of Tibetan with liver cirrhosis group including TLC9-TLC10.

10.7717/peerj.12142/supp-19 Supplemental Information 19 Raw data of Tibetan with liver cirrhosis group including TLC11-TLC12.

10.7717/peerj.12142/supp-20 Supplemental Information 20 Raw data of Tibetan with liver cirrhosis group including TLC13-TLC14.

10.7717/peerj.12142/supp-21 Supplemental Information 21 Raw data of Tibetan with liver cirrhosis group including TLC15-TLC16.

10.7717/peerj.12142/supp-22 Supplemental Information 22 Raw data of Tibetan with liver cirrhosis group including TLC17-TLC18.

10.7717/peerj.12142/supp-23 Supplemental Information 23 Raw data of Tibetan with liver cirrhosis group including TLC19-TLC20.

10.7717/peerj.12142/supp-24 Supplemental Information 24 Raw data of SRA metadata acc.

10.7717/peerj.12142/supp-25 Supplemental Information 25 Raw data of clinical basic information of all subjects.

## Additional Information and Declarations

### DNA Deposition

The following information was supplied regarding the deposition of DNA sequences: The 16S rDNA sequences are available at the Sequence Read Archive: PRJNA603295.

### Data Availability

The following information was supplied regarding data availability: The raw measurements are available in the Supplementary Files. The raw data shows all OTUs detected in this study. These data were used for subsequent statistical analysis to compare differences among groups.
