# Supplementary figures and images for: Compositional distinction of gut microbiota between Han Chinese and Tibetan populations with liver cirrhosis
Source: PeerJ. 2021 Sep 15;9:e12142. doi: 10.7717/peerj.12142 (PMC8449536; doi:10.7717/peerj.12142)

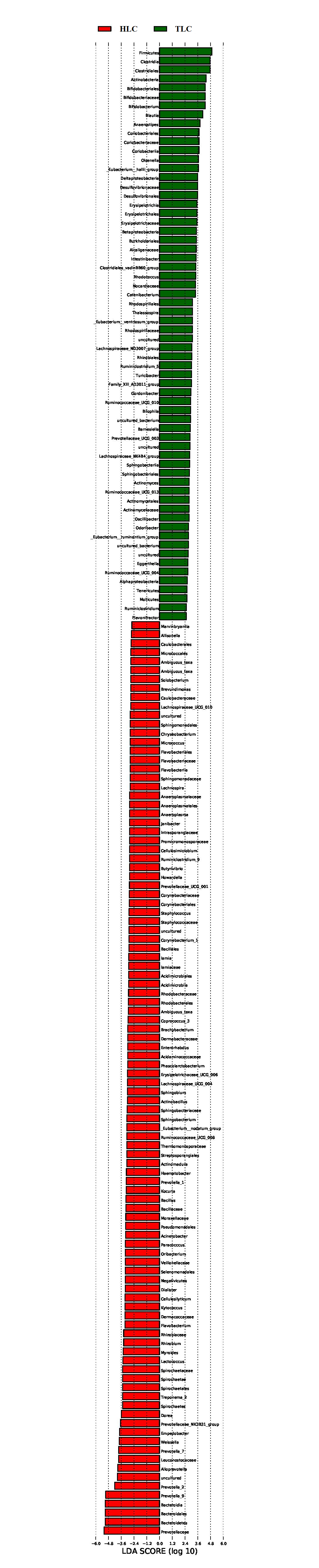

Supplement: Supplemental Information 1 [file peerj-09-12142-s001.png]
