# Supplementary material for: Compositional distinction of gut microbiota between Han Chinese and Tibetan populations with liver cirrhosis
Source: PeerJ. 2021 Sep 15;9:e12142. doi: 10.7717/peerj.12142 (PMC8449536; doi:10.7717/peerj.12142)
Supplement: Supplemental Information 2 [file peerj-09-12142-s002.docx]

**Table S1. Sequencing Data Summary**

| **Group** | **Clean sequences** | **Valid sequences** | **Mean lengths of valid sequences** | **OTU counts** | **Total OTUs** | **Valid percent**  **(%)** |
| --- | --- | --- | --- | --- | --- | --- |
| THC | 50770 | 39053 | 429 | 333 | 1579 | 74.47 |
| HHC | 19914 | 15143 | 431 | 372 | 1579 | 77.14 |
| TLC | 48338 | 36872 | 428 | 340 | 1579 | 76.76 |
| HLC | 19963 | 15006 | 431 | 408 | 1579 | 71.77 |
